# Current strategies for treatment of intervertebral disc degeneration: substitution and regeneration possibilities

**DOI:** 10.1186/s40824-017-0106-6

**Published:** 2017-10-23

**Authors:** Sebastião van Uden, Joana Silva-Correia, Joaquim Miguel Oliveira, Rui Luís Reis

**Affiliations:** 1 3B’s Research Group—Biomaterials, Biodegradables and Biomimetics, University of Minho, Headquarters of the European Institute of Excellence on Tissue Engineering and Regenerative Medicine, AvePark, Parque de Ciência e Tecnologia, Zona Industrial da Gandra, 4805-017 Barco GMR Gandra, Portugal; 2 ICVS/3B’s—PT Government Associate Laboratory, Guimarães, Braga Portugal; 3 The Discoveries Centre for Regenerative and Precision Medicine, Headquarters at University of Minho, Avepark, 4805-017 Barco Guimarães, Portugal; 4 Present Address: Bioengineering Laboratories Srl, Viale Brianza 8, Meda, Italy; 5 Present Address: Politecnico di Milano, Piazza Leonardo da Vinci, 32 Milan, Italy

**Keywords:** Intervertebral disc, Regenerative strategies, Tissue engineering

## Abstract

**Background:**

Intervertebral disc degeneration has an annual worldwide socioeconomic impact masked as low back pain of over 70 billion euros. This disease has a high prevalence over the working age class, which raises the socioeconomic impact over the years. Acute physical trauma or prolonged intervertebral disc mistreatment triggers a biochemical negative tendency of catabolic-anabolic balance that progress to a chronic degeneration disease. Current biomedical treatments are not only ineffective in the long-run, but can also cause degeneration to spread to adjacent intervertebral discs. Regenerative strategies are desperately needed in the clinics, such as: minimal invasive nucleus pulposus or annulus fibrosus treatments, total disc replacement, and cartilaginous endplates decalcification.

**Main body:**

Herein, it is reviewed the state-of-the-art of intervertebral disc regeneration strategies from the perspective of cells, scaffolds, or constructs, including both popular and unique tissue engineering approaches. The premises for cell type and origin selection or even absence of cells is being explored. Choice of several raw materials and scaffold fabrication methods are evaluated. Extensive studies have been developed for fully regeneration of the annulus fibrosus and nucleus pulposus, together or separately, with a long set of different rationales already reported. Recent works show promising biomaterials and processing methods applied to intervertebral disc substitutive or regenerative strategies. Facing the abundance of studies presented in the literature aiming intervertebral disc regeneration it is interesting to observe how cartilaginous endplates have been extensively neglected, being this a major source of nutrients and water supply for the whole disc.

**Conclusion:**

Several innovative avenues for tackling intervertebral disc degeneration are being reported – from acellular to cellular approaches, but the cartilaginous endplates regeneration strategies remain unaddressed. Interestingly, patient-specific approaches show great promise in respecting patient anatomy and thus allow quicker translation to the clinics in the near future.

## Background

The intervertebral disc (IVD) can be subjected to a great range of changes throughout a person’s life [1]. An underlying IVD degeneration (IDD) might be developing alongside with these age-related changes. IDD can be triggered by a single event of acute overloading, such as the lifting of a heavy object. It could alternatively be derived from a long-term repetitive IVD mistreatment without providing the proper conditions and time for the tissue to recover. With aging, this disease can be induced with a single event of acute over-loading of progressively less intensity. IDD may progress to serious condition due to important physiological changes that can include: Water loss, healthy extracellular matrix (ECM) synthesis decrease and phenotype change, increased cell senescence, and several other modifications at the biomolecular level [2, 3]. Ultimately, such biological transformations can lead to severe morphological changes, expressed in the form of pathologies.

The biomechanical functioning of IVD relies on a balance between the three main tissues that compose it: Two cartilaginous endplates – hyaline-like tissue located at the edge of the neighbour vertebras, the nucleus pulposus (NP) – gelatinous tissue at the centre of the IVD, and the outer and inner annulus fibrosus (AF) – two partially concentric strong elastic-like tissues surrounding the whole IVD [4]. The latter two, although slightly different from each other, will be regarded in this review as one AF when its division is not required, for simplicity purposes.

The AF, as a whole, behaves like a strong elastic material. This, however, could be a reductionist view of this tissue, since this behaviour could be derived from a low concentration of strategically located elastic fibres that convey recoil properties to the collagen fibre bundles, returning these to their pre-stressed dimensions when relaxed. Biomechanics is strongly influenced by the NP’s water concentration. Water presence is, by its turn, directly related with the biochemical composition of the tissue. In this respect, proteoglycans (PG’s) specifically play a major role in this water-biochemistry relation, due to their extremely hydrophilic nature. PG’s are responsible for the presence of up to 80% water concentration within a young and healthy NP [5]. When the IVD is compressed, the water molecules are released from the PG’s, following the lines of mechanical tension that progress from the cartilaginous endplates through the NP until the outer edges of the AF. After the loading cycle is finished water is again attracted towards the PG’s at the centre of the IVD, through diffusion derived from the closest blood vessels. In healthy IVDs, this near vascularisation is located at the cartilaginous endplates, which provide hydration as well as nutrition to the whole IVD [6]. With aging, the cartilaginous endplates progressively lose permeability due to calcification, cutting this essential water supply. However, due to the complexity of the pathways of water, nutrients, waste and oxygen within the whole IVD, the literature is not absolutely conclusive [7]. It seems that along the progressive shutdown of the endplates’ pathway, the water concentration gradually decreases within the IVD. This might force the water to return to the NP through the AF influencing a non-healthy region homeostasis with debilitated water, nutrient and waste renewability. It is clear, nevertheless, that it leads to: NP ECM remodelling unbalance, IVD loss of hydration, as well as height decrease, and abnormal force distribution. Ultimately, all these changes are responsible for the appearance of IDD morphological signs [8].

**Fig. 1:**
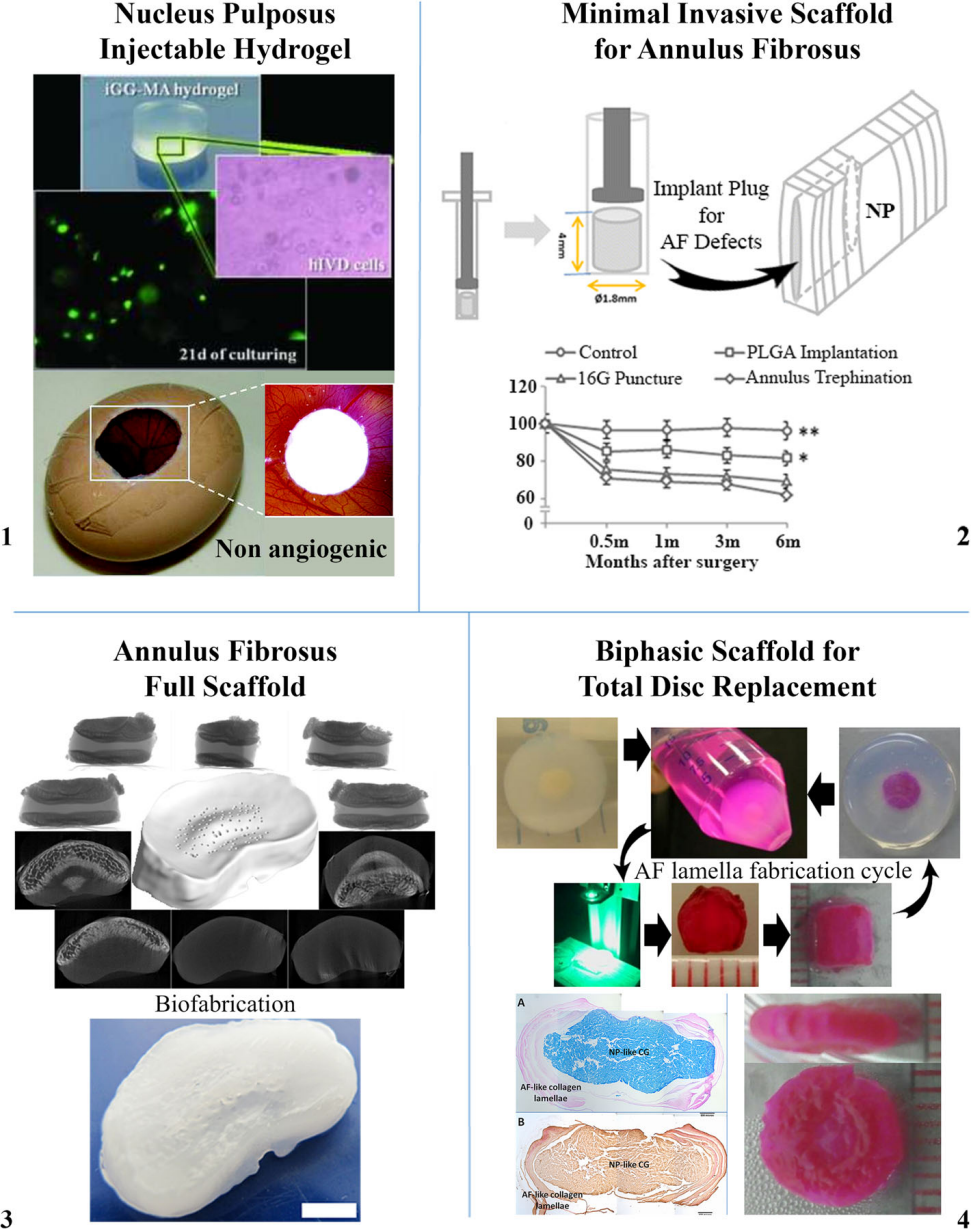
Image bundle of current state-of-the-art research strategies to treat IDD, described in this review, such as (1) injectable NP hydrogel, (2) minimal invasive scaffold for AF scaffold, (3) full AF scaffold, and (4) biphasic scaffold for total IVD replacement. Permissions: 1 – images used in this scheme were adapted from two articles of Silva-Correia et al*.* [62, 81]; 2 – images used in this scheme were adapted from Xin et al*.* [93] under the terms of the Creative Commons Attribution License (https://creativecommons.org/licenses/by/4.0/); 3 – images used in this scheme were adapted from van Uden et al*.* [99] © IOP Publishing. Reproduced with permission. All rights reserved; 4 – images used in this scheme were adapted from Choy et al*.* [95] under the terms of the Creative Commons Attribution License (https://creativecommons.org/licenses/by/4.0/)

The fundamentals related to the biological and molecular changes derived from an IVD under degeneration are described. The set of prospective and recent studies that have been reported, ranging from biomaterials-based to cellular approaches, are also herein overviewed (Fig. 1).

### Intervertebral disc degeneration (IDD): biological and molecular changes

Biological and molecular changes underlie the morphological signs of IDD. Several cellular changes occur during IDD, namely in: Type, concentration, death, proliferation, senescence, and phenotype. All these changes have a non-healthy impact on the biochemical synthesis and its consequent composition, which leads to an unbalanced ECM remodelling, and ultimately to hydration loss [9].

The IVD is composed of different types of cells and cell type concentrations in its different tissues. The cartilaginous endplates, in resemblance to the hyaline cartilage, are composed of chondrocytes. The elongated fibroblast-like cells can be found in the outer part of the AF, while the inner part of the AF has more rounded chondrocyte-like cells. The cell population in these tissues does not change significantly with aging. However, it can greatly vary within the NP [1]. The cartilaginous endplates and the whole AF, as most of the spinal structures, are derived from the mesoderm germ layer, but not the NP [10]. The NP originates in the endoderm germ layer, which is also the notochordal cell’s origin. It is believed, that the young NP tissue does not have the chondrocyte-like cells, but only notochordal cells, that disappear by the end of the first decade of life [11]. The notochordal cells are gradually replaced by chondrocyte-like cells, which are believed to migrate from the inner AF, from the cartilaginous endplates, or both [12]. There is, however, some controversy around the cell concentration changes within the NP. Zhao et al. [1] reported that cell concentration has its turnovers in parallel with the change of cell type predominance. As notochordal cells start to decrease, the cellular concentration significantly declines creating a positive feedback for chondrocyte-like cells to migrate and proliferate into the NP. These cells gain concentration along with IDD progression. Bae et al. [13], however, claims that the degenerated IVD cellular concentration is lower than in healthy IVDs due to gradual loss of optimal cellular environment conditions. Both hypotheses present strong arguments, possibly indicating a wide variability of possible IVD aging and degeneration progressions in humans.

**Fig. 2:**
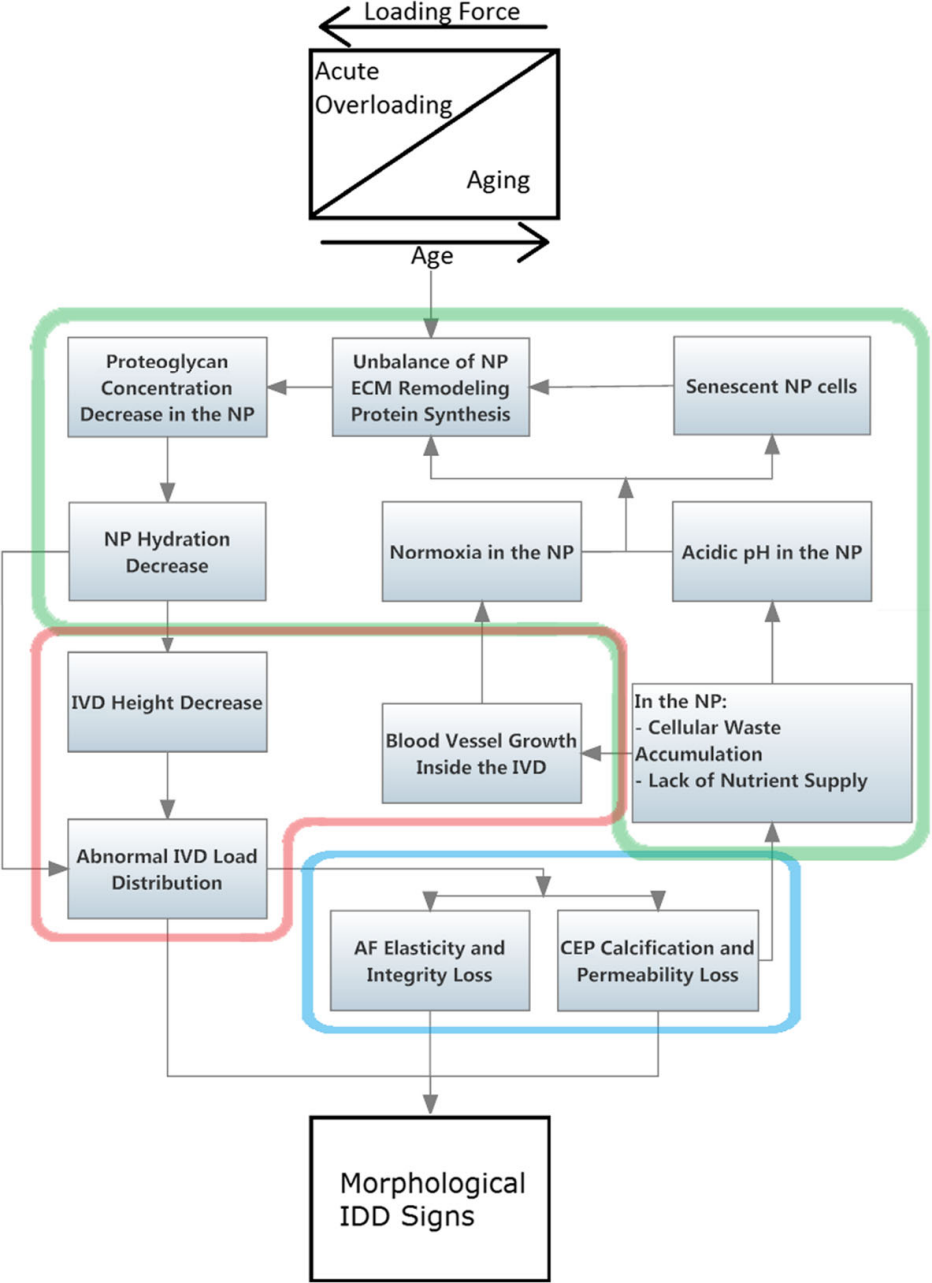
Cascade of events associated with IDD morphological signs. Starting by a contribution of an acute or a repeated set of acute loading forces that, as tissue aging progresses, becomes lighter to set IDD. The highlighted areas divide the events by tissue/groups of tissue – Red: IVD; Green: NP; Blue: AF and cartilaginous endplates. NP: nucleus pulposus; AF: annulus fibrosus; IVD: intervertebral disc; CEP: cartilaginous endplates; IDD: IVD degeneration disease

Cellular environment progressively changes due to scarcity of nutrition and waste removal pathways as cartilaginous endplates calcify, causing initial cell death (Fig. 2) [14]. With IDD progression, ingrowth of blood vessels accompanied by nerve growth occurs into the AF continuing into the NP, in a later stage [15]. This tends to increase nutritional accessibility and waste removal rates, which by its turn can provide conditions to increase cellular concentration within the NP. With this neo-vascularisation proximity, however, also the oxygen concentration rises, leading to overall biochemical environment towards a normoxic NP environment that diverges from a healthy IVD [16]. NP cells’ phenotype and activity is stimulated by hypoxia. Ultimately, a prolonged excessive availability of oxygen leads these cells to a senescent state, which negatively impacts long term cellular concentration [17, 18].

**Fig. 3:**
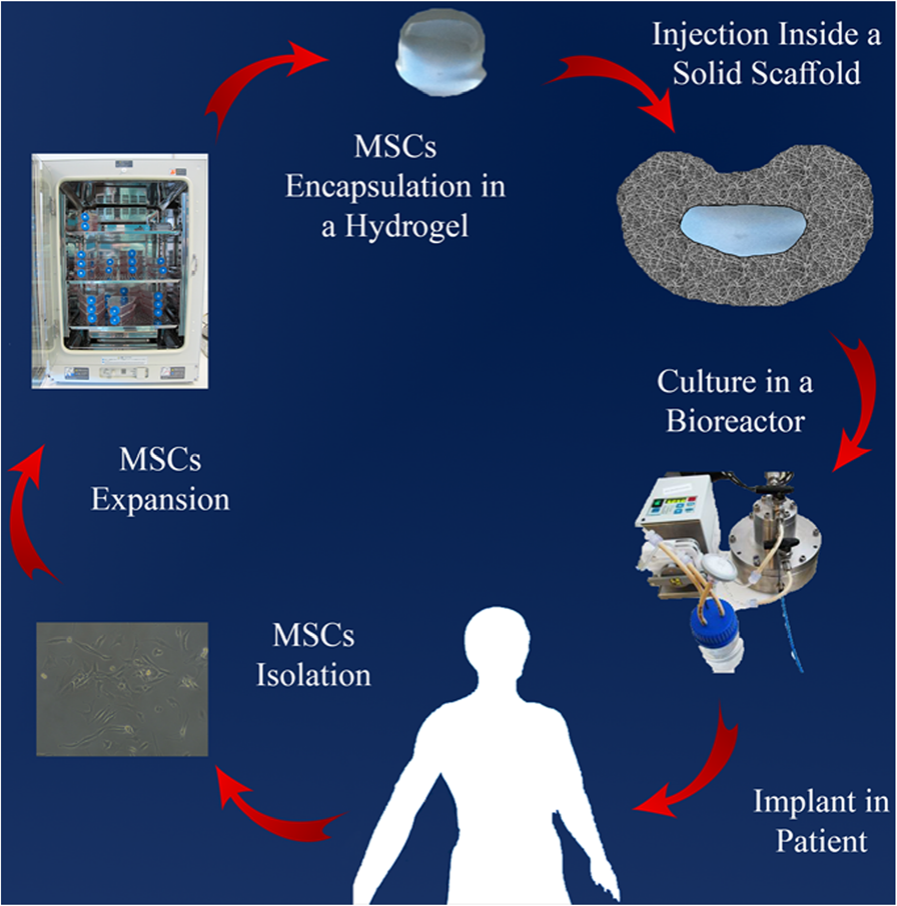
Scheme of a tissue engineering strategy applied to the IVD

**Fig. 4:**
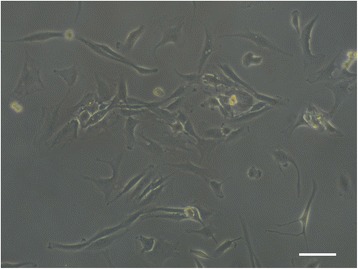
Micrograph of human NP cells after 3 days in culture. Scale bar: 50 μm

**Fig. 5:**
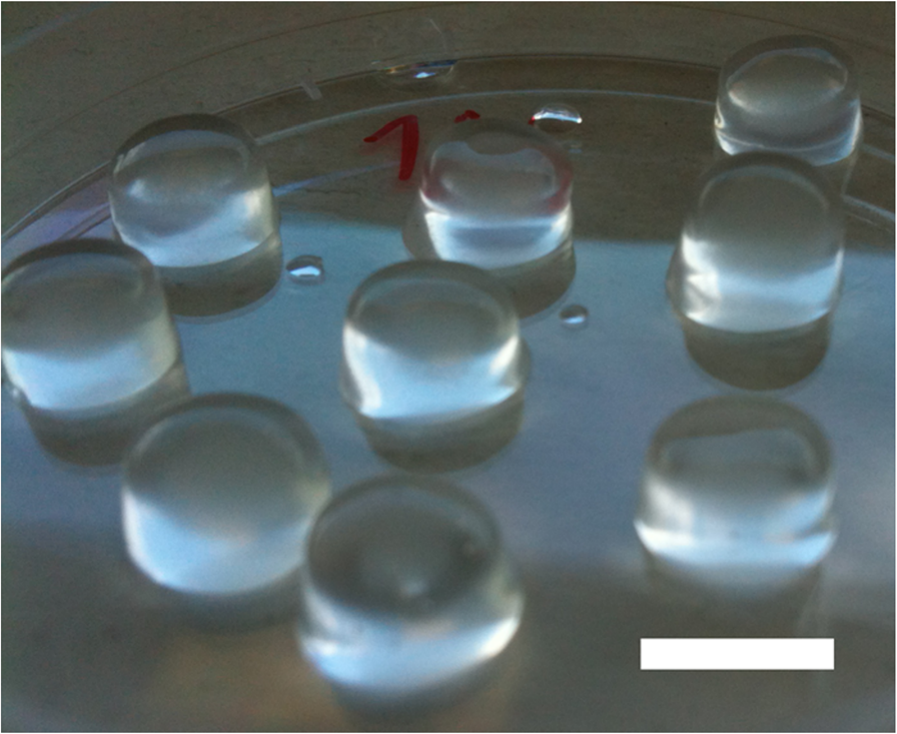
Methacrylated gellan gum discs with a diameter of 10 mm and a height of 5 mm. Scale bar: 10 mm

**Fig. 6:**
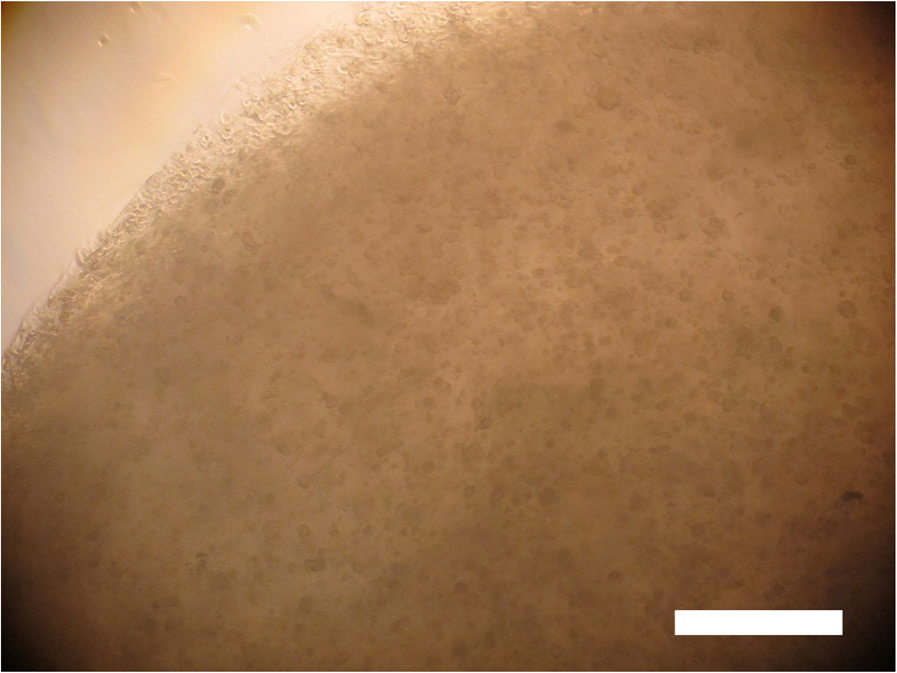
Micrograph of methacrylated gellan gum hydrogel with one million rabbit NP cells encapsulated, after overnight culturing. Scale bar: 200 μm

**Fig. 7:**
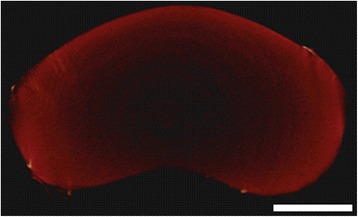
Micro computed tomography image top view of the rabbit AF, surrounding a dark area, which is the NP. Acquisition parameters: pixel size – 13.18 μm, source – 89 kV and 112 μA. Scale bar: 250 μm

**Fig. 8:**
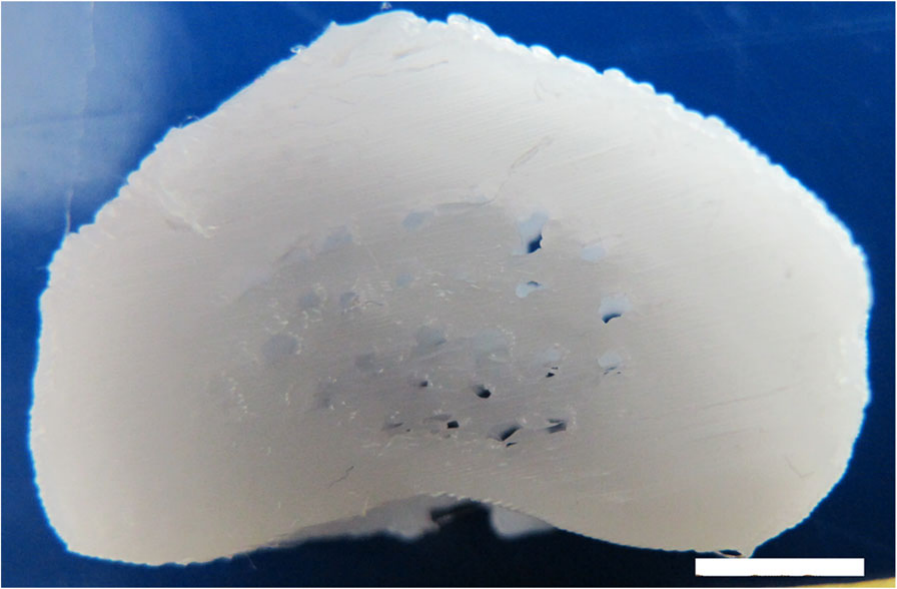
Photograph of a 3D printed PCL rabbit IVD replica. Scale bar: 5 mm

A healthy AF is largely composed of collagen type I whereas the NP is mainly composed of collagen type II and aggrecan [1]. Other molecules are also present in the NP at low concentration, namely: Fibronectin, collagen type I, III, V, VI, IX and XI, and other types of PG’s, such as: Biglycans, decorin and fibromodulin [9]. In healthy IVD, there is also a production of catabolic molecules with increased expression while degeneration develops. These molecules are matrix metalloproteinases and aggrecanases, responsible for breaking down the ECM to allow its natural remodelling cycle. Matrix metalloprotainases-1, 2, 3, 7, 8 and 13 are expressed in the NP. In addition, there is also an increased cytokine production, such as: Tumour necrosis factor-α, interleukin-1α and -1β. These molecules also promote matrix metalloproteinases’ synthesis, which in degenerated IVDs can reach levels that have a devastating effect over the ECM [19]. Zhao et al. have summarized the biochemical changes caused by IDD, which complements this brief description of matrix remodelling mediation [1].

The phenotype change undergone by NP cells is possibly the highest responsible factor, within the NP, for the morphological transformations derived from IDD. This shift in cell expression influences directly the hydrophilic anabolic-catabolic ECM balance. Overall, IVD hydration decreases while more matrix is degraded than it is produced, ultimately leading to IVD hardening and unhealthy biomechanical behaviour [18].

NP hydraulic permeability greatly depends on the magnitude of compression force requested by the IVD. Heneghan et al*.* [20] defined a mathematical formula explaining this phenomenon, which is given by equation 1.

1 $$ k\left(\lambda \right)=1.59\times {10}^{-15}{\left(\frac{\lambda -0.2}{0.8}\right)}^{1.13}{e}^{\left[-\frac{0.02\left({\lambda}^2-1\right)}{2}\right]} $$

The function of IVD permeability is given by k in relation to the stretch ratio λ, corresponding to the ratio between the compressed sample’s height (h) and the original uncompressed height (h_0_). On the apparatus used by this research group, a human IVD is submitted to a certain compression strain, while the inlet and outlet flows are measured. This experiment describes an exponential relation between permeability and compression (0 ≤ λ ≤ 1). Interestingly, until a certain magnitude of compression (λ ≤ 0.2) the tissue is not permeable, possibly implying the need for IVD loading cycles to create an optimal micro-environment [20]. However, this formula can only be applied when the NP is healthy, when the degeneration starts the PG number decreases and the permeability increases together with it, *i.e.* the water retention decreases and the whole IVD decreases its biomechanical performance. A regular loading becomes an overloading, which also becomes a factor to progress further on the degeneration state that will in the end reduce even more the mechanical capacity, and the cycle continuous following a recessive spiral [21, 22].

### Prospective strategies for intervertebral disc (IVD) regeneration

In accordance with the severity of the IDD, different treatment strategies can be applied. These can be invasive or not, and pharmacological or not. A Swedish massage or acupuncture, for example, is a non-pharmacological and non-invasive treatment commonly used for managing low back pain, a symptom that is strongly related with IDD [23]. Pharmacological treatments are also used for acute strong pain expressivity, such as lumbar muscle spasm commonly managed with muscle relaxant. However, non-invasive treatments have a limit and do not help resolving more severe conditions, in which repair strategies such as spinal fusion or screws implantation must be applied. These, are very effective in eliminating the pain, but reduce spine flexibility [24]. Additionally, due to the mechanical properties’ discrepancy between the implants and the biological tissues, local trauma can produce dramatic repercussions. If that is not the case, long term presence of these devices in the spine alters progressively the biological properties of adjacent tissues, possibly inducing degeneration of neighbour IVDs [24].

Advanced regenerative strategies, currently under research, aim for total IVD regeneration by addressing all previous signs of degeneration, maintaining the treated IVD in homeostasis with its environment. Tissue engineering is a clinically innovative driving force focused on this objective.

The gold standard diagnostic tool for IDD is magnetic resonance imaging (MRI). The degeneration-associated morphological changes observed, using the equipment, are more evident on the NP than for the AF or the cartilaginous endplates [25, 26]. Possibly, for this reason, the tissue engineering’s initial IVD research efforts were carried out to address the huge challenge of NP regeneration (Fig. 3). Cell-loaded hydrogel injection solutions were initially envisioned to regenerate the NP, believing this could be the pivot for a total IVD regeneration. Some cases can benefit from this minimally invasive intervention, mostly light to medium severity IDD conditions. In advanced states, however, the AF has lost its mechanical integrity to support a full NP’s volume and its highly hydrated consistency, creating a tendency for herniation [27]. Research strategies for the combined regeneration of the AF and NP started to become more common. However, results were not as effective as possibly predicted. It is conceivable that ignoring the essential role of the cartilaginous endplates is the missing piece for a successful regeneration strategy.

The tissues that compose the IVD are closely related, working symbiotically to maintain the whole IVD healthy and functional. If one tissue is left degenerated, the applied regenerative strategy, although demonstrating short to medium term positive results, will probably fail in the long run. The cartilaginous endplate nutrition and waste removal pathway needs to be re-established for a complete IVD healthy homeostasis. Nonetheless, without a vigorous NP hydrophilic matrix, water retention remains scarce, and this pathway remains blocked or minimized, and the hidden tissue regeneration potential is not stimulated as a whole – the NP needs to be addressed as well. Yet again, if the AF does not have the capability to withhold the NP, the material used for regenerating the NP will herniate outside the IVD, possibly causing low back pain. With this rationale, we return to the beginning of the regeneration strategies’ loop, indicating that every degenerated IVD tissue should be treated for the whole strategy to succeed. Extensive innovative efforts for NP and AF regeneration have been investigated, and the most important examples are herein discussed. Cartilaginous endplates’ targeted regeneration seems to be the missing link that will enable their full success.

### Nucleus pulposus (NP)

When aiming to regenerate the NP, the water content must be addressed if the biochemical environment has been compromised. Hydrophilic materials, such as hydrogels are used to substitute the water uptake responsibility, while newly implanted cells have time to produce native PG-based ECM. If, however, the native matrix is still mildly rich in hydrophilic molecules a cell-based strategy alone, carried by a non-polymerizable carrier might be enough.

Cell-based strategies aimed to regenerate the IVD concern strictly on increasing the NP cell concentration to renew biochemical environment by synthesis of ECM. This is addressed by cell injection not only to increase cell number but, more importantly, to boost the active cellular population [28, 29]. Since, as aforementioned, the matrix is gradually lost due to the native cells’ change in behaviour, which seize to produce PG’s at an even rate as the remodelling proteins catalyse the ECM [13]. The cells of a NP with advanced state of degeneration are senescent and need to be replaced with resilient and vigorous ECM producers.

#### Cells for nucleus pulposus (NP) regeneration

Cell therapy approaches for IVD regeneration are based on injecting NP-like cells, responsible for synthesizing PG’s when in situ. Therefore, the first decision factor is - what is the right type of cells to be used? If native NP cells (Fig. 4) need to be replaced they surely cannot be used, due to their senescence or diseased phenotype expression [30, 31]. Fortunately, it is possible for this to be a wrong argument in the near future, since Abbott et al*.* [32] have been working on increasing senescent human NP cells metabolic activity, proliferation, glycosaminoglycan production, and stimulate non-degenerated phenotype. They believe that native cells from a NP in a severe state of degeneration have, in fact, a regenerative potential that can be explored. Exposing this type of cells to a cocktail of specific growth factors, to notochordal cells’ conditioned medium, or both, constitutes a promising strategy to enhance glycosaminoglycan production phenotype as a means for an effective treatment. In their study, they retrieved cells from a human source. After expansion, under the three aforementioned conditions, cells were implanted in rabbit models with induced IDD (induced by needle-punctures). Results demonstrated not only that cells stayed viable for up to 24 weeks, but also that this strategy delayed the degeneration progression [32]. A specific follow-up of this work would be very interesting to increase scientific information on the strategy’s potential. Nonetheless, work being developed by these authors has been very revelling, *i.e.* by Iatridis, Purmessur and co-workers, such as the work on the effect of notochordal conditioned media and its derived factors in inhibiting vessel and nerve growth [33, 34].

The use of notochordal cells to be implanted in degenerated IVDs is also being considered as a cell-based strategy, whether from stem cell-derived allogeneic or even xenogeneic origin, or from autologous origin, based on specific differentiation protocols. However, as Arkesteijn et al*.* observed [35], there was no change in anabolic response within the *ex vivo* NP culture when the xenogeneic notochordal cells were implanted (porcine cells in bovine NP). Additionally, seeded notochordal cells did not display a native morphology by the end of the culture period – 42 days. A different conclusion from the observations reported by other studies [36, 37], possibly due to a non-realistic clinical approach regarding a very high number of notochordal cells used in the other studies, they say. A discrepancy in ratio of notochordal cells in relation to native NP cells of 20:80 instead of 50:50 might be the reason for the lack of success [35]. Possibly unrealistic clinical approaches, although good at extending scientific knowledge, might not be able to be directly used as a patient treatment, but can certainly provide a basis for other strategies that will.

Properties of notochordal cells’ conditioned medium have also been in the line of research of Bach et al*.* [38], whose research led to the conclusion that notochordal cells condition medium derived from human, canine and porcine, had a regenerative effect on human chondrocyte-like cells (herein named as NP cells for simplicity purposes). Meaning that human notochordal condition medium is not required to achieve that effect, since canine or porcine have an approximate effect [38]. Porcine notochordal cells conditioned medium can, therefore, be used for different treatment strategy purposes, due to its potential for extensive availability. Autologous senescent NP cells could be isolated and cultured with this conditioned medium and be delivered back to the diseased IVD. Differentiated autologous stem cells could be cultured in this medium to boost their maximum hydrophilic matrix synthesis potential before implantation.

The prospect of using stem cells in cell-based strategies for IVD regeneration brings with it the concern of how to guide differentiation into fully functional NP cells. Stem cell research efforts (*e.g.* induced pluripotent stem cells) [39] have been helping to reduce the obstacle of stem cell number availability. Concerning specifically the differentiation of stem cells into NP cells, the initial number of cells required significantly depends on the method that is being followed. It is feasible, in principle, either in vitro or in situ.

The in vitro differentiation method has the advantage of assuring that the implanted cells have the optimal phenotype, though, with the disadvantage of more stem cells being required. Additionally, stem cell expansion is not trivial, and many cells are lost while differentiating into NP cells [40]. The key factor, in IVD regeneration is hypoxia (2% O_2_), since this is the environment that NP cells are acquainted. In vivo, these cells can be up to 2–3 mm away from the closest blood vessel [17, 18]. Therefore, the most promising strategy to ensure that stem cells differentiate into metabolically active NP cells might be to culture them in a hypoxic environment. However, a lot of stem cells tend to die due to the lack of oxygen, requiring even more stem cells at start.

Fang and his co-workers [40] have been studying the hypothesis of manipulating mesenchymal stem cells in order to make them resistant to hypoxia by adding an anti-apoptotic gene called B-cell lymphoma-2. By avoiding stem cells to suffer apoptosis at the early stage of differentiation, pre-chondrocyte-like phenotype cells were produced to be resistant to low concentrations of oxygen, thus being able to maintain cell numbers under this condition. Half of the number of stem cells needed was used. Nevertheless, a serious doubt remains – does this not increase the chance of cancer cell formation? Manipulating directly stem cells by decreasing their ability to resort to apoptosis is a step towards cells being unable to commit apoptosis, and losing the ability to die, which would make them virtually cancer cells. Nevertheless, this work brings great promise and, if proven safe, there is no reason why this method cannot be applied as a treatment strategy for IVD regeneration.

In situ differentiation method has the advantage of using a standard approach for stem cell expansion. Leckie and co-workers [28] developed a work following this type of cell-based method. Its purpose was to determine whether injecting human umbilical tissue-derived cells into the NP would improve the course of IDD. The cells were injected with phosphate buffer solution, EVICEL®-based carrier alone and cell-laden EVICEL®-based carrier. Briefly, EVICEL® is a combination of a fibrinogen-based solution with a thrombin-based solution, both derived from human plasma. Follow-up was based on MRI, biomechanics and histologic findings. The results were significantly away from the positive control (non-punctured IVDs), and failed to fully restore MRI signs for non-degenerated IVDs, possibly due to the unclear choice of carrier. A different hydrogel might have produced significantly more interesting results. However, they were successful in slowing down the degeneration process and showed better results than the negative control (punctured IVDs). At 12 weeks, the MRI results showed that cells alone and cells delivered using EVICEL®-based carriers were significantly distinct from punctured values. Regarding the viscoelastic properties, the cell-free carrier and cells in EVICEL®-based carrier were significantly closer to positive control (non-punctured IVDs) than the cells alone. However, if the biological and biochemical conditions within this tissue are not modelled, the use of this method could stimulate the growth of blood vessels and nerve endings inside the NP. In the literature, there are evidences that the appearance of blood vessels and nerve endings inside the NP might be originated by native stem cells [41]. Therefore, the injection of more stem cells without a modulated differentiation pathway in a diseased environment could promote this, as well as deteriorate the state of the tissue by further degenerating it.

#### Processing of scaffolds for the nucleus pulposus (NP)

When the NP biochemical environment has no conditions to maintain a healthy cellular activity, a highly hydrophilic material should be implanted. This material, typically a hydrogel, is meant to create suitable conditions for cells to express healthy chondrocyte-like phenotype. With time, native hydrophilic ECM is produced while the hydrogel degrades, ideally, at an even rate. Therefore, when choosing the right biomaterial to mimic a healthy NP cellular environment, several properties must be considered, as described in Table 1 [5, 42–46].

**Table 1:** Overview on the NP treatment approach from the materials engineering perspective

	Problem	Solution	References
1	Accessing the NP is only possible through the AF, leaving fissures that increase probability of herniation appearance.	Injectable material – so that only the area of the needle’s section of the AF is wounded.	[42]
2	The material cannot be polymerised before implantation, if it needs to be injected.	Material able to polymerize in vivo, by pH, ion interaction, temperature, light, or another possible factor.	[43, 44]
3	The NP tissue of a degenerated IVD tends to be a hostile environment for cells.	The material’s mechanical properties should be as close as possible with the mechanical properties of a healthy NP tissue. Hence, re-establishing IVD height and biomechanical function.	[45]
4	Cells injected in the NP of degenerated IVDs tend to lose the desired phenotype, due to the lack of hydrophilic molecules.	The material must be able to absorb a lot of water, at least 80%, just as a healthy NP matrix.	[5]
5	Non-degradable materials do not create or limit the space for new healthy tissue to grow.	The degradability rate must match the tissue’s rate of regeneration, not being at the expense of the mechanical properties.	[46]

Considering all the properties in Table 1, hydrogels stand out as an ideal candidate material (Fig. 5). Hydrogels are polymeric networks with the capacity to absorb water from 10 up to 100 times its dry weight. [47]. Several kinds of hydrogels, natural and synthetic, have been studied for IVD tissue engineering strategies [48].

There is a growing interest in natural-origin hydrogels that are able to be processed but not synthesised, which significantly reduces production costs. Other reasons for why they are becoming more attractive might be their low level of cytotoxicity, their wide range of possible tissue engineering applications, as well as astonishing biological properties, such as: Bioactivity and bioactive degradation, while available for cellular remodelling and cell adherence [49–51].

Although hydrogels from natural-origin offer a wide range of biological advantages, it might be difficult to find the desired range of physical properties. Nonetheless, know-how on these materials is widening, new materials are being studied and new processes are being developed to tune the right properties [47, 52]. The IVD has a slow metabolism, which leads to long regeneration time. The degradation rate of the hydrogel to be implanted needs to match this time frame [53]. Some natural-origin hydrogels have been proposed in the literature. The hydrogels that seem to be more popular for NP regeneration are based on the following raw materials: Alginate [54–56], chitosan [48, 51, 57, 58], collagen [51, 59, 60], gellan gum [48, 49, 61–65], and hyaluronic acid [48, 51, 57, 66] (Table 2 [48, 49, 51, 54–60, 66, 67]).

**Table 2:** Summary on hydrogels applied in IVD tissue engineering research

*Material*		*Advantages*	*Disadvantages*	*References*
*Natural Origin Hydrogels*	Alginate	• Polymerization under mild conditions; • Injectable in situ; • NP similar mechanical properties; • Cell adherent.	• Lack of long-term mechanical stability; • Impurities make it unpredictable; • Difficult to sterilize and to handle.	[54–56]
	Hyaluronan	• Non-immunogenic; • Easy control over the polymer chain sizes; • Bioactive; • Low manufacturing cost.	• Osteogenic; • Cytotoxic in high concentration.	[48, 49, 51, 66]
	Chitosan	• Bioactive; • Cell adherent • Antibacterial activity; • Non-immunogenic.	• Bad mechanical properties; • Cytotoxic cross-linkers; • Impurities make it unpredictable.	[48, 49, 51, 57, 58, 67]
	Collagen	• Non-immunogenic; • Piezoelectric properties; • Bioactive.	• Bad mechanical properties; • High degradation rate; • Some level of toxicity (cross-linking agents).	[48, 49, 51, 59, 60]
	Gellan Gum	• Non-angiogenic; • Able to polymerize until 1% (w/v); • Non-immunogenic; • Very low manufacturing cost; • Good mechanical properties.	• Weak in physiological conditions due to the exchange of divalent cations by monovalent ones.	[48, 49, 61–65]
*Synthetic Hydrogels*	Polyethylene glycol	• pH-switchable electronic properties; • Photo-polymerizable; • Adjustable mechanical properties; • Easy control over architecture and chemical composition.	• Bioinert; • No cell adherence; • Expensive to manufacture.	[48, 74, 138, 139]
	Polyvinyl Alcohol	• Catalytic activity; • Increases viscosity when added to other hydrogels; • Controllable crystallinity.	• Bad mechanical properties; • Regular chain structure; • Non-degradable; • Expensive to manufacture.	[48, 67, 138]
	Polyvinyl-pyrrolidone	• Good mechanical properties; • Biocompatible.	• Non-degradable; • Expensive to manufacture.	[51, 72, 74]

In alternative, synthetic hydrogels provide predictable and reproducible chemical and physical properties that might be tuned for different tissue engineering applications, *e.g.* degradation rate according to the aimed tissue regeneration rate. Moreover, they easily blend with polymers that broaden even more the properties possibilities [68]. Since they are made of well-known molecules, when pure, they have a low risk of immunogenicity, infection and toxicity [51, 69]. Though, they lack bioactivity that is characteristic of natural-origin materials, and their manufacturing process is, in general, economically less attractive, which are two key disabling factors for a tissue engineering application. Hence, while natural-origin materials lack diversity of properties, synthetic materials lack options that are economically viable. Some examples of synthetic hydrogels being applied in NP tissue engineering strategies are: Polyethylene glycol [27, 70], polyvinyl alcohol [71, 72] and polyvinylpyrrolidone [51, 73, 74] (Table 2). There are, indeed, not many studies using just synthetic-based materials for NP regeneration. The interesting possibility, however, is to use a natural-based material modified with synthetic polymers to achieve the advantages of both types [56, 60].

#### Combined therapy: cell-seeded scaffolds for nucleus pulposus (NP) regeneration

A self-assembling peptide called RADA16-I [Ac-(RADA)_4_-CONH_2_], with the ability to form hydrogels, was proposed by Tao et al*.* [75] as cell carrier for NP tissue engineering. Interesting predisposition of this material to form β-sheet configurations when within water was reported. Due to its C-terminus, it is possible to conjugate this peptide with various bioactive short-peptide motifs. The conjugation of three different short peptides of bone morphogenetic proteins-7 was evaluated [75], and a 1:1 ratio of RADA-KPSS [AC-(RADA)_4_-GG-KPSSAPTQLN-CONH_2_] with RADA16-I, named RADA-KPS, was found as the optimal functional formulation to promote proliferation and activate, in vitro, degenerated NP cells to express collagen II and aggrecan. Wu et al*.* [76] further demonstrated that bone marrow mesenchymal stem cells, encapsulated within RADA-KPS, had a time-related increase in expression of factors typical of metabolically active NP cells – collagen II and aggrecan.

The idea of using collagen II hydrogels for NP regeneration is natural, since it is one of its most prevalent ECM components, when healthy [1]. Although this material has a very high cost, its advantages might compensate for that matter. Tao et al*.* [59] demonstrated that collagen II hydrogel formulations with higher concentration increase the tendency of initially seeded adipose derived stem cells to express a NP cell-like phenotype. However, collagen II on its own does not seem to possess the required mechanical properties for NP tissue engineering, a cross-linker or a material to blend seems to be required [77]. From the same research group, Zhou et al*.* [60] used N,N-(3-dimethylaminopropyl)-N’-ethyl carbodiimide together with N-hydroxysuccinimide, which according to their literature search are non-cytotoxic. In their study, it was evaluated the proliferation and differentiation effect of this cross-link formula on collagen II hydrogels with encapsulated adipose derived stem cells. Cell proliferation almost doubled from 7 to 14 days of cultures, such as the control – same cell type on tissue culture polystyrene substrate, demonstrating no cytocompatibility change due to cross-linking. The substantial positive evidence lays on the gene expression profiles, since the control seems to show no significant difference on the typical NP cells’ phenotype profile between 7 and 14 days of culture. While for the cross-linked collagen II hydrogels, all gene expressions analysed increased about 20-50% over the non-cross-linked on both time-points, with a slight drop difference on the collagen I expression – a non-typical ECM molecule of healthy NP. Continuing comparing these results with the non-cross-linked collagen II hydrogels, the gene profile expression was more interesting but the increase in rate of expression was practically the same. Demonstrating that it is possible to maintain a good differentiation profile, while achieving higher mechanical properties with this cross-link formula.

Gellan gum material (Fig. 6) was first proposed by Oliveira et al*.* [78–80] for cartilage tissue regeneration. Cytocompatibility towards chondrocytes was demonstrated as well as the ability to polymerize with both temperature and pH change. Silva-Correia et al*.* [47], however, first specifically proposed its application for NP regeneration. In several studies the gellan gum’s properties were extensively characterized, such as: Rheology [63], biocompatibility [81], and non-angiogenic potential [62]. Pereira et al*.* [82] proposed the use of gellan gum microparticles within a gellan gum matrix with different ratios of high- or low-acyl gellan gum. An interesting rationale considering the cellular capsules’ configuration typically observed in native NP [83]. Moreover, the independent reactions of glycidyl methacrylate and methyl benzoylformate, reported by this research group, demonstrated the versatility of this material to be tuned to acquire stronger mechanical properties and ultra-violet light polymerization [84]. In contrast with methacrylation reaction, an alternative to increase gellan gum’s mechanical properties was developed by Thorvaldsson et al*.*, who proposed an interesting rationale to develop hydrogels of that same material reinforced with electrospun polycaprolactone (PCL) nanofibers. Therefore, mimicking the natural molecular organization and morphology of native NP ECM. The scaffold was developed by combining PCL electrospinning with gellan gum air brush spraying on a rotating collector partially immersed in calcium chloride polymerizing solution [85]. Expectation is high to understand if the remaining non-evaporated chloroform-methanol solvent residues, used on the electrospinning side, are below the cytotoxicity threshold for cells loaded in the gellan gum solution when both reach the collector. In addition, the polymerization time of gellan gum in ionic solution is very rapid, a characteristic of interest to be applied in contemporary fabrication technologies, such as bioprinting. More recent works propose, as well, the possibility for gellan gum to be applied in IVD regeneration [64, 65], corroborating its potential for this application.

Alongside gellan gum is the carboxymethylcellulose, another polysaccharide suggested for NP tissue engineering, in this case by Reza and Nicoll [86, 87]. As gellan gum, carboxymethylcellulose can also be methacrylated, acquiring the photo-polymerisation ability. However, its application for NP degeneration treatment has only been proposed in the methacrylated form [88–90], due to the reversible nature of conventional ionic cross-linking techniques [86]. Since 2009/10, this research team has extensively characterised methacrylated carboxymethylcellulose material’s properties, namely: Mechanical, swelling ratio, and diffusion, with and without seeded cells [86, 88, 90]. Reza et al*.* [87] analysed the ECM development and functional properties of NP cells within this hydrogel. They compared two different types of culture medium - the standard serum-containing medium formulation versus a serum-free chemically defined medium, both supplemented with transforming growth factor-β3. Glycosaminoglycan and collagen II content was significantly greater in serum-free constructs, as well as the Young’s modulus, and the equilibrium weight-swelling ratio of the same constructs approached that of the native NP tissue. The results showed why the use of a chemically defined medium is so relevant, undoubtedly weighted by its high cost, therefore, although pertinent, it does not indicate that it is economically viable. More recently, the differentiation profile of mesenchymal stem cells into NP-like cells was studied, within this material, based on the transforming growth factor-β3’s rate of exposure and cross-linking density. It was reported that low molecular weight carboxymethylcellulose with low methacrylation and monomer concentration, provides more promising results for NP tissue engineering [89]. On another perspective, the continuous supplement of transforming growth factor-β3 although stimulating cells to more interesting results, might not compensate its significantly higher cost in relation to a transient supplement. Transient exposure rate seems enough to reach biochemical and mechanical levels of construct maturation observed in native NP [88].

To overcome the increased cost of growth factors and chemically defined serum culture medium for stem cell differentiation towards NP phenotype, as well as overall construct maturation, Thorpe et al*.* [91] cultured human mesenchymal stem cells in 5% oxygen conditions within a hydrogel of poly(N-isopropylacrylamide)-(N,N’-dimethylacrylamide)-Laponite® – shortly named as pNIPAM-DMAc-Laponite®. Results shown expression of native NP-like phenotypic markers and ECM. Therefore, by not using a chondrogenic inducing medium or supplemented growth factors the regeneration strategy is simplified [91] and has a lower cost.

### Annulus fibrosus (AF)

An optimal treatment for the AF (Fig. 7), aiming its full regeneration, depends largely on its state of degeneration. A mildly degenerated AF having a localized tissue damage might be treated with a minimal repair intervention [92–94], which can be sufficient to bring the whole AF to a complete healthy condition. However, engineering strategies reported in the literature describe that in case the AF, as a whole, is compromised a total IVD replacement is required, *i.e.* including the NP [95–97]. Considering the biphasic nature of a young healthy IVD [95], it seems promising to mimic those material properties on both phases. Research for a biphasic total IVD replacement can, naturally, be developed for both tissues at the same time or strictly focused on the AF analogue. With such a high attention by the research community on NP regeneration throughout the last decade, as aforementioned, there are very promising strategies that could be coupled in the development of a biphasic scaffold for total IVD regeneration.

The AF connects both cartilaginous endplates and, together with them, surrounds the NP. Each one of the cartilaginous endplates is connected to one of the two adjacent vertebras [4]. Therefore, if the AF is going to be replaced it needs to be with a scaffold that maintains a functional structure while providing the conditions for peripheral ECM to develop across the border between the AF and the cartilaginous endplates, before biomaterial degradation. The IVD’s metabolism is relatively slow, probably due to their low vascularity nature [98]. Selecting the appropriate material to develop an AF replacement scaffold will probably have to respect that time frame, to achieve a positive long-term outcome.

#### Cells for annulus fibrosus (AF) regeneration

Unlike the NP, the cellular aspect of AF regeneration strategies described in the literature is not as complex or, at least, not explored to the same extent. In summary, an AF scaffold can be developed mainly via three different possibilities: Acellular [99], AF cells [97], or stem cells [100].

AF cells are classified as fibroblast-like [101], and fibroblasts are one of the easiest primary cell types, if not the most, to culture in vitro. In fact, usually they are unwanted, since sometimes they contaminate the isolated cell culture due to their higher rate of proliferation, representing the worst and most common type of autologous contamination in cell isolation procedures [102]. This proliferative advantage is, in this case, a benefit that can be explored. Whether through the easiness to isolate and expand native AF cells, which already have the desired phenotype, or by following an acellular scaffold strategy and rely on native taxis of fibroblasts or AF cells (in the case of minimal intervention) from adjacent tissue(s). Acellular strategies reduce critical procedure steps, costs and time significantly when compared with seeding scaffold approaches. It has the potential to be, in practical terms, off-the-shelf.

Total IVD replacement strategies, encompassing a cellular AF-like scaffold, are required to focus on achieving a final multi-type cellular population. This includes NP and AF cells, as well as respective progenitor cells to maintain their concentrations within the desired regions long after the patient’s intervention. Perhaps for this reason, stem cells are viewed as an interesting cell source to seed annular scaffolds, since the same cells can be used to seed the NP scaffold, while maintaining the potentiality to differentiate into the respective aimed phenotypes.

Recent research indicates that the most popular stem cell types for AF regeneration are mesenchymal stem cells [94, 103] and AF-derived stem cells [100, 104]. Valadà et al*.* made a very comprehensive review on stem cell sources for IVD regeneration, including: Muscle-derived, olfactory, induced pluripotent, hematopoietic, synovial, embryonic and Wharton’s jelly [105], however, it was not distinguished the application of each stem cell type forAF or NP.

There is, however, who believes it might be interesting to explore cell injection therapy for AF regeneration. Freeman et al*.* claimed, to their knowledge, to be the first to follow that strategy [106]. They started by inducing IDD on ovine models, using a method they previously described [107], which comprises a postero-lateral annulotomy incision on the left side. This was followed by 3 days of post-operative recovery and 6 months of IDD maturation, confirmed by MRI. A postero-lateral injection of one million allogeneic bone-derived mesenchymal stem cells, isolated from the iliac crest, was further administrated on the opposite side of the lesion. MRI was made after 3 and 6 months, while animals were sacrificed at the latter time point for a biochemical and histological analyses of each IVD. Disc height index results expressed a continued recovery until the last time-point, when its index equalizes the positive control group, which did not suffer annulotomy and received a phosphate buffer saline injection. It is interesting to add that the negative control, *i.e.* untreated degenerated IVDs, kept a constant index between 6 and 12 months, meaning that all annulotomy IVDs at 6 months were at the lowest level of disc height index. This study, however, is limited to the type of IDD originated by physical damage to the AF. Additionally, it is not possible to infer if this strategy would reverse IDD derived from cartilaginous endplates calcification [7, 14]. Nonetheless, the results demonstrate the importance of further exploring a more biological and less material-oriented approach for AF regeneration [106].

#### Processing of scaffolds for the annulus fibrosus (AF)

The AF varies greatly along its radius, as a consequence, perhaps, part of the literature on the subject makes a distinction between inner and outer AF [82, 108]. The AF and NP are actually a biocomposite structure with no clear evident borders. Its biochemistry seems to change gradually as well as significantly along its radius – from the centre of the NP to the most peripheral layer of the outer AF. It is not easy to mimic the complex arrangement of the ECM molecules, as if it was a continuous medium, to form the macro-structure of the IVD. The most promising way might be to produce a whole IVD construct by observing the IVD as a discrete assembly of few regions. There is at least one different type of ECM component with a concentration that prevails in each one of the three structures [5]. In opposition is the biochemical and morphological composition of the outer AF. This anisotropic tissue [109] is mostly made of collagen I, making it an extremely elastic tissue [95]. The inner AF, although having more collagenII than the other two types of tissue, is like a mixture of the other two in terms of mechanics and biology, while having a morphological organisation closer to the outer AF [110].

Several biomaterials have been developed and used as prime-material for preparing scaffolds to address AF regeneration. Figure 8 shows a typical AF shape that was replicated by 3D printing PCL based on a reverse engineered rabbit IVD micro-computed tomography (micro-CT) acquisition [99]. Other interesting biomaterials proposed on the literature are described in the form of a significant number of different composites and formulations, based on the same prime-materials, as: Polylactic-co-glycolic acid [93], silk fibroin [111], collagen [95, 112], and PCL [97, 99, 113, 114].

As aforementioned, the IVD is an anatomical structure highly subjected to complex movements and strains [115]. If a construct without pre-maturation is envisioned for implantation, the material chosen to replace the AF, in the short-term, must be able to withstand that biomechanical responsibility until neo-ECM is able to do it. It should have a similar Young’s modulus that enables the material to sustain high forces while remaining free from plastic deformation. In the long-term, it must degrade at the same rate that cells synthesize the neo-ECM.

ECM synthesis and remodelling depends on the biochemical balance existing inside the cell construct. The piezoelectric properties (*i.e.* conversion of mechanical force into electrical signals, and vice-versa) of collagen molecules, present within it, possibly play an important role in this process, dependant on the constant cyclic loads applied to the IVD [116]. The lines of tension change dynamically according with this load cycles, resulting in degradation stimulation of the AF scaffold and synthesis of new ECM, which probably affects piezoelectric effect inside the structure. Cells are attracted to where pressure is felt, since the collagen upon mechanical stimulation releases electrical stimulus that attract and stimulate cells to produce more ECM, for the whole tissue to be mechanically balanced. This effect is similar to what happens in almost every tissue of the human body. Bone is a good example, since trabeculae and its mineralization are coincident with tension lines felt by the tissue [117]. Scaffolds with piezoelectric properties, such as collagen-based scaffolds for the AF, have a head start advantage. In fact, constructs with no piezoelectric properties, when implanted, probably have a cell taxis disadvantage in relation to surrounding native tissues. In summary, the way that the scaffolds degrade, and how its mechanical properties vary with it, is important for the cell construct to be successful upon implantation.

The matrix of the AF is mainly made of collagen, being the principal reason why several research groups have been proposing it for annular scaffold preparation [112, 118, 119]. Scaffolds made of collagen I or II have already shown to stimulate IVD cells to produce big PG’s and long glycosaminoglycan chains [119, 120]. Choy et al*.* developed a strategy to produce biphasic scaffolds composed of mechanically tuned collagen and glycosaminoglycan molecules, with collagen-glycosaminoglycan modifications and photochemical-cross-linked collagen membrane previously developed in separate by their group [121, 122]. An interesting layer-by-layer approach was established to progressively prepare the AF scaffold surrounding the NP replacing collagen-glycosaminoglycan core, this way mimicking the native AF layered morphology. Each layer is produced via the following cycle – the core is encapsulated in the modified collagen solution and further removed for photo-cross-linking, it is then dehydrated by rolling it over a filter paper and rehydrated again to create a phase-border between the next layer, which starts by repeating the first step [95].

PCL has been proposed for numerous biomedical applications due to its chemical versatility [123], easy processability and tuning possibility for matching its mechanical properties to the desired role [124]. In particular, PCL has a larger elastic domain than other candidate materials for AF [125], due to its low melting-point of approximately 60°C [126]. In a recent work at our group, a 3D printed scaffold made of PCL was developed using a custom-tailored geometry of a rabbit AF, acquired by micro-CT and segmented 3D modelling [99]. The thermoplastic properties of this material allow it to be processed by fused deposition modelling 3D printing, providing all the possibilities that this technology offers. Cultured AF cells with leachables extracted from those PCL scaffolds did not develop a cytotoxic effect on AF cells [99]. A follow-up work was developed that progresses the proof-of-concept of a custom-tailored scaffold biofabrication to a patient-specific type. Oner et al*.* [127] used PCL to 3D print a patient-specific AF using MRI data acquired within a clinical environment. This contrasts with the previous work that used a micro-CT system, which provides significantly more data allowing the development of a 3D model replica with equally higher fidelity to the original scanned tissue. However, neither CT nor MRI standard clinical imaging systems have this level of resolution. Demonstrating the increased need for the clinical environment to keep up with equipment requirements of immerging treatment strategies. Wismer et al*.* [113] studied the ability of PCL material to support AF tissue regeneration and its interaction with its native cells. It was demonstrated that AF cells could proliferate in electrospun oriented PCL sheets, and that the ECM produced was rich in glycosaminoglycans [113]. Other studies have also reported similar findings regarding the compatibility of PCL with AF cells [128, 129].

Minimal invasive treatments of AF defects, in its turn, are an approach that carries less risks than a tissue engineering total IVD replacement approach. Its certification and application in the clinics would be probably easier and fits well with standard procedures, like discectomy. Facing an IVD herniation, the surgeon can decide to remove the herniated NP tissue and use a suturing approach to close the damaged region of the AF [130, 131]. Regenerated strategies are being developed to repair the AF defect and stimulate its regeneration [92, 93]. Xin et al*.* developed small cylindrical scaffold plugs, with a diameter of 1.8 mm, made of polylactic-co-glycolic acid containing gelatine particles with a diameter range of 280 to 450 μm. After leaching, a scaffold is produced with an average pore size within the same diameter range of the gelatine particles. The plugs were implanted in rabbit models of AF degeneration, and a successful repair of the AF defect was verified with an overall degeneration regression [93].

Polytrimethylene carbonate material is generating interest to fabricate space-fillers for AF defects [92, 94, 132]. Long et al*.* [92] developed a composite repair strategy that combines a conical space-filler composed of polytrimethylene carbonate secured in place by fibrin-genipin adhesive sealant, previously tuned to match the shear properties of native AF tissue [130]. To completely ensure the non-extrusion of the implant, a polyurethane membrane patch is also applied over the implant surrounding the native tissue. The implantation was carried using previously extracted bovine coccygeal region-of-motion IVDs, which were further submitted to biomechanical assessment. However, the membrane was not enough to eliminate the risk of implant extrusion. Interestingly, the results showed an increased performance for the application of only fibrin-genipin, which decreased the re-herniation risk to low levels. It is less clear, however, after surpassing the risk of re-herniation, if the polytrimethylene carbonate would not achieve better tissue regeneration results in the long run.

#### Combined therapy: cell-seeded scaffolds for annulus fibrosus (AF) regeneration

The main advantages of acellular strategies are related to its simplicity of production and regulatory point of views, storage simplicity, sterilization process and low cost. Ideally, as soon as the scaffold is implanted the degradation time should start. As previously mentioned, the biomechanical balance can only be compensated by the ECM synthesis. Cellular strategies prevail when this goal is mainly considered, since cells can start producing matrix as soon as they are seeded and stably attached. Making taxis of migrating fibroblasts, from surrounding environment to the cell construct, synergic instead of fundamental. It is not clear, however, what approach would achieve more interesting results in the long run, since there are advantages and disadvantages on both sides, as herein reported. Further in vivo studies on both approaches are required.

The application of urethane-based polymers for AF is a very interesting idea, due to its very elastic properties. Nonetheless, a series studies developed last year by Li et al*.* use a polyether-carbonate-urethane-urea to electrospun AF scaffolds, with the promise of being biodegradable [100, 103, 104]. The work by Zhu et al*.*, in particular, encompassed a degradation assay, [100], which was obtained following the synthesis protocol of the polymer, while demonstrating its non-cytotoxic character [133]. What is interesting, however, is the use of AF-derived stem cells as the cell source. Liu et al*.* demonstrated that aligned electrospun fibres increase the AF cell phenotype expression in comparison with random orientation [104]. Zhu et al*.* tuned several types of polyether-carbonate-urethane-urea for different elastic profiles and its effect on AF stem cell’s gene expression was evaluated [100]. The authors reported that collagen I expression increased alongside the material’s elasticity, while collagen II and aggrecan had a contrary relation.

A biphasic scaffold composed of lamellar regenerated silk fibroin surrounding a fibrin-hyaluronic acid NP, seeded with AF cells and chondrocytes, respectively, was reported by Park et al*.* [111] following a biomimetic approach. After silk fibroin dissolution, a sodium alginate solution was added and injected into cylindrical shaped silicon moulds. The assemblies were subjected to freeze-drying and further crystallized in water for 6 hours to generate β-sheet formation within the silk fibroin. The alginate was removed from the scaffold by immersion in water for 24 hours and the toroidal-shaped silk fibroin scaffolds were achieved by simple punching. The results showed dense lamellar structures with spaces between lamellas varying from 10 to 400 μm. Cell seeding was more efficient and homogeneous throughout the scaffold than in their previous study, where a porous silk fibroin scaffold instead of lamellar was produced [134]. The morphological structure of the silk fibroin toroidal IVDs seemed to guide collagen deposition very efficiently [111]. Maybe, with a culture time longer than two weeks, the cell constructs could obtain the desired mechanical properties, since silk fibroin is strong but not elastic, therefore, with further deposition of collagen the cell construct could become elastic. Until now, unfortunately, it does not seem to exist a follow up of this work, which would be interesting.

IVD tissue engineering research is already extensive and several obstacles are being surpassed, though there is an argument not typically addressed, which is the structural compatibility, not in terms of morphology but in terms of optimal fitting within the intervertebral space. The AF is often mimicked by a toroidal shape scaffold, with not even an IVD-like geometry. Even if the IVD-like shape is mimicked, the top and down surfaces are not prepared according to the endplates’ surfaces, which have unique topographies in each person, especially in diseased IVDs. Therefore, creating a high potential for displacement, once implanted, upon cyclic spine loading. This was recently addressed, by creating a fused deposition modelling 3D printed AF scaffold, in which its computer aided design was a result of 3D modelling based on a real IVD imaging dataset [99]. Briefly, the micro computed tomographic acquisition focused on the spinal motion segment. During segmentation, the osteochondral borders between the IVD and the adjacent vertebras were carefully followed as the top and down edges of the IVD model, therefore creating a full replica of the original IVD and providing the potential for a precise fitting within the intervertebral space.

An alternative for secure implantation between adjacent vertebras is to follow, for example, what is already done with spinal fusion, by removing the IVD and joining the two flat vertebral surfaces together creating one continuous double vertebra [135]. Instead of joining those surfaces, however, it might be also possible to introduce a tissue-engineered spinal motion segment that possess top and down osteochondral layers, leaving exposed a bone-like surface that can attach to the native sectioned vertebras. Therefore, in line with the strategy developed by Choy et al*.* [95], described in the previous section, Chik et al*.*, envisioned a scaffold that mimics an entire spinal motion segment [136]. This composite construct is a full IVD assembly of NP-, AF-, and endplate-like tissues. It employs a strategy previously described by the same research team, based on micro encapsulation of mesenchymal stem cells in collagen I to produce microspheres, named naive subunits. These are used in two separate differentiating mediums – chondrogenic and osteogenic. At the end of the third week, both layers are assembled together with an additional thin layer of collagen and mesenchymal stem cells in between [137]. The NP core is also prepared following the same method they previously described [121], based on collagen and glycosaminoglycans. The NP core was placed between two osteochondral units with both chondral layers facing the NP. These assembly was then processed following the same strategy described by Choy et al*.* [95], which allows the layer-by-layer encapsulation and polymerization of photochemically-cross-linked collagen I. In this work, however, mesenchymal stem cells were added between each collagen layer, as well as in the NP core. This study served as a proof-of-concept for this complex strategy, naturally with that many steps and details, some tuning is still required, since the construct is not yet able to match the maturity of a native spinal motion segment in several aspects. Considering the amount of collagen, glycosaminoglycans, and mesenchymal stem cells are required to produce the whole structure. The costs of applying such strategy, as a treatment, are probably extremely high. However, it is our understanding that no one has ever been able to replicate so faithfully a native IVD in vitro [136].

## Conclusions

Reviewing the literature on tissue engineering strategies for IVD regeneration it is possible to state that extensive efforts have been made to regenerate the NP alone, but increasingly for the simultaneous NP and AF has been addressed. Two of these strategies have stirred the research community focused on IVD regeneration: In vivo and ex vivo approaches that balance risk of adverse effects with treatment efficiency, with the first being potentially fatal but more efficient.

In tissue engineering field, the literature seems to be in consensus about using a polysaccharide as a low-cost NP substituting material. Additionally, due to the high avascular nature of this tissue a tight compromise is in order – the degradable biomaterial must allow cell adhesion and proliferation, while not hampering for angiogenesis to occur. Gellan gum seems to fulfil these requirements, having already demonstrated its non-angiogenic potential, as well as providing an optimal environment for chondrocyte culturing, both in vitro and in vivo. Collagen II, as a more expensive alternative to polysaccharides, has shown excellent results in vitro regarding NP native-like ECM production, possibly aided by its natural piezoelectric properties when under mechanical stimulated culture conditions. It would be interesting, perhaps, to assess the combination of gellan gum doped with collagen II to accomplish the positive results of both materials. Nonetheless, if a patient also requires AF regeneration, the NP substituting hydrogel needs to be integrated in an AF scaffold. Several approaches for integrating both tissue regeneration substitutes have been reported, being integrated from the beginning of construct preparation or only just before application. PCL has been very popular due to its mechanical properties, having been processed in several different manners with interesting final results. It has, in fact, also been applied within the biofabrication field, showing the possibility to produce 3D printed custom-tailored AF scaffolds based on the host intervertebral geometry. This way further expanding processability into a wider range of scaffold shapes and details, with low-cost and in a short time. Patient-specific therapies are becoming popular and allow the development of scaffolds or constructs that fit the patient’s needs in a precise manner. This increases the potential for construct integration into the surrounding tissues, and lowers the risk of complications, such as scaffold displacement or mechanical destabilization.

Although the recent reports serve the great purpose of providing tools to regenerate a greater tissue volume of the IVD, few strategies have been developed to regenerate the cartilaginous endplates. The healthiness of these plates is, in fact, one of the keys for a healthy cellular environment within the whole IVD. Regenerating the other two tissues, alone or together, when the endplates are calcified is, therefore, a desperately frustrating task, ultimately leading towards failure in the long-run. The strong bonds between the three tissues that compose the IVD, on all levels, imply that the tissue engineering path of less resistance is a strategy that encompasses all tissues together, when considering late-stage IDD cases. Having into account what has been developed for NP and AF regeneration leads to the conclusion that once a successful cartilaginous endplate regeneration strategy has been developed the remaining strategies will possibly achieve significantly better results when applied together. However, full IDD is not the only clinical condition of this disease. Research on minimally invasive surgical approaches and repair/regeneration strategies that rely on the use of patient-specific implants, for the AF and NP, play an important role in finding appropriate clinical solutions for less severe, but still painful, conditions of IDD.

## Abbreviations

IVD: Intervertebral disc
IDD: Intervertebral disc degeneration
ECM: Extracellular matrix
NP: Nucleus pulposus
AF: Annulus fibrosus
PG: Proteoglycan
MRI: Magnetic resonance imaging
PCL: Polycaprolactone
CT: Computed tomography
